# Youth Advocates' Perceptions of Tobacco Industry Marketing Influences on Adolescent Smoking: Can They See the Signs?

**DOI:** 10.3934/publichealth.2016.1.83

**Published:** 2016-03-04

**Authors:** Malinda Douglas, Andie Chan, Marilyn Sampilo

**Affiliations:** 1 Oklahoma State Department of Health, Office of the State Epidemiologist, 1000 NE 10th St, Oklahoma City, OK 73117, USA; 2 Oklahoma State Department of Health, Office of the Tribal Liaison, 1000 NE 10th St, Oklahoma City, OK 73117, USA; 3 University of Oklahoma Health Sciences Center, Department of Pediatrics, 1100 N. Lindsay, Oklahoma City, OK 73104, USA

**Keywords:** tobacco, POS advertisements, youth, advocacy, empowerment, adolescent development

## Abstract

Point-of-sale (POS) advertising at retail stores is one of the key marketing avenues used by the tobacco industry. The United States Surgeon General urges actions to eliminate POS tobacco advertisements because of their influence on youth smoking. Many youth empowerment programs are implemented to address tobacco industry marketing influences, including POS tobacco advertisements. While youth are asked to take on such collective action, little is known regarding their perceptions and understanding of tobacco industry marketing influences and related advocacy activities. This mixed methods study examined Oklahoma's tobacco control youth empowerment program members' perceptions of tobacco industry marketing influences. Four focus groups were held with active program members from rural and urban areas. Overall, the focus group participants viewed the program as purposeful, as an avenue to help others, and as a way to make a difference. Specifically, the older participants (median age = 18 years) identified tobacco industry marketing influences such as POS, movies, and magazine advertisements and reported participating in activities that counter POS tobacco advertisements at retail stores. Likewise younger participants (median age = 16 years), identified similar tobacco industry marketing influences, but also included tobacco use by friends and family as tobacco industry marketing influences. Moreover, the younger participants did not report engaging in activities that addressed POS tobacco advertisements. The study results suggest that the empowerment program should tailor its programming, training, materials, and activities with input from youth of various ages. Thoughtfully developed messages and specific activities can truly empower youth and maximize their contribution as change agents who address POS or other initiatives at the retail environments to prevent chronic diseases.

## 1. Introduction

The tobacco industry spends billions of US dollars each year to market their products at retail stores through tobacco displays, brand signs, and price promotions, also known as point-of-sale (POS) tobacco advertisements [1]. Consequently, youth are widely exposed to POS tobacco advertisements, particularly in low income neighborhoods and within close proximity of schools [2]–[5]. Exposure to POS tobacco advertisements influences youths' positive perception of tobacco, smoking susceptibility, smoking initiation, and brand loyalty [6]–[10]. With nearly 90% of smokers trying their first cigarettes by the age of 18, the United States Surgeon General has urged communities to take action supporting policies, programs, and media campaigns to prevent youth tobacco use initiation [11].

Vast investments have been made in youth empowerment programs to engage youth as change agents to expose tobacco industry marketing influences. Drawing upon their own experiences and perspectives of tobacco industry marketing influences, youth tell their story and call for actions. Several counter-tobacco industry campaigns conducted by youth have been quite successful in reducing youth smoking and promoting changes within the communities [12]–[13]. Such youth empowerment programs were designed to address youths' lack of knowledge and sense of control to generate prevention alternatives and action [14]. Modeled after American Legacy Foundation's Statewide Youth Movement Against Tobacco Use program [15], the Oklahoma tobacco control youth empowerment program was created with a mission to empower and unite youth to resist and expose big tobacco's lies while changing current attitudes about tobacco. The Oklahoma program consisted of local youth teams located mostly at schools and coordinated by full- and part-time staff. The program's mission was integrated into training and program materials and communicated via a network of staff at the state, regional, and county levels. Community awareness events, assessment of POS tobacco advertisements with promotion of its removal, checks of retailers' compliance with tobacco purchasing laws, anti-tobacco sponsorship demonstrations, and meetings with community leaders were noted in program materials as activity options to enable the team members to affect their environment.

While previous evaluation studies tested empowerment theories or policy-passing abilities [16], relatively little is known about the youths' perceptions and their impact on youth empowerment programs. As a part of comprehensive knowledge management and translation effort [17], a mission-focused mixed methods study assessed the Oklahoma youth empowerment program's team members' perceptions and experiences of: 1) the program, 2) tobacco industry marketing influences on adolescent smoking, and 3) program activities. Understanding the youth members' perceptions of these three areas and applying what was learned with existing empowerment theories can enhance youth programming that addresses chronic disease prevention at the retail environment level.

## 2. Methods

This mixed methods study combined qualitative and quantitative data collected during focus groups. Each group was conducted purposefully with fewer participants and for only 60 minutes since team members were adolescents [18]. Adapted from the American Legacy Foundation's survey instruments, a structured discussion guide of 10 questions with supporting questions and prompts and brief participant questionnaire were developed and tested with program staff and others to ensure a smooth question path, unnoticed transitions, and the use of terminology familiar to team members [18]–[19]. The key questions are shown in Table 1. The study protocol was approved independently by the Oklahoma State Health Department and the University of Oklahoma Health Sciences Center Institutional Review Boards.

**Table 1.** Key questions for focus group discussion.

1) How does this program differ from other youth organizations that you know of?
2) What one word describes the program?
3) What are three ways the tobacco industry influences youth smoking or spit tobacco use?
4) How are decisions made about what activities the team does?

The program's administrative databases of 210 program staff and volunteers and 17 local tobacco control programs were reviewed to identify active youth teams that were: 1) composed of members aged 13 to 18 years of age; 2) associated with local tobacco control programs; 3) supported by training, technical assistance, activity guides, advocacy opportunities, and other support from program staff and adult volunteers; and 4) meeting on a regular basis. After removing administrative (n = 19), inactive (n = 69), duplicated (n = 23), nonresponsive (n = 30), and unassociated (n = 12) staff, the 57 identified teams were categorized based upon the population of the county where each team was located as urban (150,000 and greater population) or rural (< 150,000 population). Two teams were selected randomly from the eight urban teams and two teams were selected randomly from the 49 rural teams for focus group participants to enhance geographic sample diversity. Among the selected teams, only youth members who had participated in at least half of the team meetings during the past twelve months were asked to participate. The selected teams had similar characteristics of all the active teams in the state (Table 2).

**Table 2.** Characteristics of teams eligible for focus groups selection.

Characteristics	Selected Teams (n = 4)	All Teams (n = 57)
Staff's length of time with team	19.5 months	21 months
Staff's experience in tobacco control	3.0 years	3.0 years
Staff's experience in youth programs	11.6 years	10.0 years
Time spent on team programming	7.8 hours per month	8.1 hours per month
Youth who participate in at least 50% of team activities	54%	60%

Program staff who regularly worked with the selected teams provided invitations and follow-up reminders to enhance recruitment efforts. Signed parental consents and youth assents were obtained prior to focus group participation. Each focus group was held at the same location, day of the week, and time as a regular team meeting. The focus groups were conducted at three high schools and one youth center in 2008. The focus group discussions were recorded by two digital audio recorders and later transcribed verbatim. A quantitative questionnaire was administered to participants at the end of each focus group.

Immediately after each focus group, the trained facilitator and co-facilitator discussed the group interaction, potential themes, and social context using field notes taken by the co-facilitator. For qualitative data analysis, the facilitators used the long table method [18]. Specifically, each facilitator independently sorted participant quotes into clusters to identify major themes within and among focus groups. Weight in the analysis was given to comments that related to the purpose of the focus group and that were represented by at least one of the following factors: frequency of comment or theme that occurred in all groups; specificity provided through telling of personal experience, intensity expressed when comment given; and extensiveness of people who expressed a similar comment. Emerging themes, major findings, and clustered quotes were compared and contrasted among the facilitators to develop a coding system and achieve consensus on study findings. Once a coding system was established, EZ-TEXT software was used to manage the qualitative data with the goal of 85% inter-rater reliability [20]. The quantitative data from the questionnaires were tabulated using SAS statistical analysis software (version 9.1). Participants received written reports after the study was completed.

## 3. Results

### 3.1. Focus group participants

Eighteen youth members participated in the four focus groups. On average, the participants were 16 years of age and in the 10th grade. One-third (33%) of the participants selected Native American as a descriptor of their race. Of those, all (100%) selected another race as well. Demographic variables, including race/ethnicity, were collected in the quantitative survey (Table 3).

**Table 3.** Demographic characteristics of participants.

Characteristics (*n* = 18)	Percentage (n)	
Gender		
Female	55.6% (10)	
Male	44.4% (8)	
Length of time in program		
< 1 month	27.8% (5)	
1 to 3 months	22.2% (4)	
4 to 6 months	0.0% (0)	
7 to 11 months	22.2% (4)	
1 to 2 years	11.1% (2)	
More than 2 years	16.7% (3)	
Age in years		
13-16 (younger)	61.1% (11)	
17-18 (older)	38.9% (7)	
Race/Ethnicity (one or more)		
White	72.2% (13)	
Native American	33.3% (6)	
African American	16.7% (3)	
Latino/Hispanic	5.6% (1)	
Other	5.6% (1)	

As observed during the focus groups, many participants wore sports uniforms or wore t-shirts from various school clubs and other participants spoke of their involvement in sports, band, and other clubs. In three of the four focus groups, participants mentioned other team members who could not attend the focus group because of work, sports, or other school activities.

### 3.2. Reasons for being in the program

The participants viewed the tobacco control youth empowerment program as different from other youth programs or clubs because it had purpose - more specifically, the purpose of changing the environment around them by influencing youth not to use tobacco. When asked what interested them in the program, themes emerged in three main areas: concern for others, making a difference, and increasing their knowledge of tobacco.

- *The other clubs are really just to have fun, but this one is to really send a message.* (Group D, male)
- *We don't want the elementary kids pick up the bad habit.* (Group D, female)
- *I have gotten a lot of insight about tobacco that I didn't know before.* (Group A, male)
- *I thought I can change the way people view tobacco companies.* (Group C, male)

As the participants described the program in one word, “life-changing” was a term mentioned during three of the four focus groups. Each participant described this word differently.

- *Well, it is like, we watched a facts movie when we first started, like, first did the first [program] thing and we watched this movie and like thing and it showed how it could give you, like, cancer and heart disease and if you want to have an early death then.* (Group B, female)
- *It changes your perspective towards it but also, like, doing all these things at all these different schools and sponsoring games and stuff, you change a lot of other people's lives, too.* (Group C, male)
- *Life-changing for the people we influence.* (Group A, female)

### 3.3. Perceptions of how the tobacco industry marketing influences youth tobacco use

During each focus group session, participants constructed a group list using individual lists of ways the tobacco industry marketing influences youth smoking or spit tobacco use. All participants agreed that tobacco industry marketing could influence youth tobacco use through magazine advertisements and product use in movies and television shows. Most of the participants agreed that POS tobacco advertisements were tobacco industry marketing influences. Half of the participants identified flavored tobacco products, tobacco packaging colors, and rodeo sponsorships as industry influences. All responses, including the uniquely identified influences, are listed in Table 4.

**Table 4.** Tobacco industry marketing influences identified by participants.

Agreement	Tobacco industry marketing influences	
**All** (4 of 4 groups listed)	Advertisements in magazines Movies Televisions shows	
**Most** (3 of 4 groups listed)	Friends and family who smoke Signs/displays in retail stores	
**Some** (2 of 4 groups listed)	Candy and other flavored tobacco products Package colors Rodeo sponsorships truth^®^	
**Few** (1 of 4 groups listed)	Billboards Giveaway booths Internet commercials Prizes from product purchases	

Two non-tobacco industry marketing influences were identified by the participants. Most of the participants erroneously included tobacco use modeling behavior (friends and family who smoke) as tobacco industry marketing influences. The only participants that did not include tobacco use by friends and family had been with the program a shorter period of time, but were on average 12–18 months older than the other participants. Some participants listed a counter-marketing campaign, truth^®^, as a tobacco industry marketing influence because it revealed that the tobacco industry was selling deadly products and lied about it.

### 3.4. Selection and participation in activities to address tobacco industry marketing influences

Most of the participants mentioned using the program materials as a reference when selecting activities for participation. Other factors influenced the selection of activities, including expectations, team processes, community connection, available resources, and novelty. Most participants stated their intentions behind activity selection and participation were to help keep younger children from starting to use tobacco and to help others quit using tobacco.

- *Just make a tobacco-free community or help with it.* (Group A, female)
- *…[W]e don't want the elementary kids to pick up the bad habit coming to junior high...thinking it's cool because others are doing it.* (Group D, male)
- *I thought we could change the way people view tobacco companies, the products, and help people to quit.* (Group C, male)

The participants' intentions to prevent children from using tobacco and to help others quit were also reflected in their responses to the quantitative survey. The participants reported they engaged in various types of activities as team members (see Table 5). Nearly all (94%) of youth were involved with team meetings and community awareness activities, such as anti-tobacco industry rallies, demonstrations, and event sponsorships. While completing the questionnaire, a few participants asked the others how to categorize tobacco cessation promotional activities. As a result, one-third (3/9, 33%) reported an anti-tobacco rally or demonstration were health promotion/education activities. Only the oldest participants reported assessing POS tobacco advertisements, testing retailer adherence to legal purchasing age, and advocating for voluntary removal of POS tobacco advertisements in retail stores. The oldest participants also accounted for the majority (3/5, 60%) of those who reported meeting with community officials.

**Table 5.** Involvement in activities and events among participants.

Activity/Event (N = 18)	Response Percentage	Response Count
Team meeting	94.4%	17
Community awareness	94.4%	17
Anti-tobacco (industry) rally or demonstration (n = 9)		
Sponsorship at school or community events (n = 8)		
Training	72.2%	13
County training/meeting (n = 6)		
Regional training/meeting (n = 4)		
State youth summit (n = 2)		
National conference (n = 1)		
Health education	61.1%	11
Health education activity other than health fair (n=8)		
Health fair (n = 3)		
Address tobacco industry at retail stores	44.4%	8
Assess POS tobacco advertisements (n = 4)		
Test retailer adherence to legal purchasing age (n = 2)		
Removal of POS tobacco advertisements (n = 2)		
Meetings with community officials	27.8%	5
Other (specified by participant)	27.7%	5
Focus group (n = 4)		
Poster (n = 1)		
Community assessment other than assess POS at retail stores	16.7%	3

## 4. Discussion

This study set out to determine if a tobacco control youth empowerment program was achieving its mission by examining its members' perceptions of the program, tobacco industry marketing influences, and participation in activities that addressed tobacco industry influences, including POS tobacco advertisements. Results demonstrated that youth participants joined the program because of their concern for others, desire for knowledge, and motivation to make a difference. They also found that it had a greater purpose than those of other clubs or programs. This is consistent with another study that found that youth who were highly involved in a similar program also were motivated to make a difference [21].

Youth participants had some difficulty identifying some forms of tobacco industry marketing and had difficulty distinguishing between tobacco industry marketing influences and tobacco use among family and friends. Yet, all teams received ongoing training, resources, and support from program staff to help increase their knowledge and understanding to expose the influence of tobacco industry marketing on youth smoking. Moreover, the younger participants perceived tobacco industry marketing influences as a blend of tobacco industry marketing (advertisements in magazines, product placements in movies and television, and POS advertisements), counter-tobacco marketing campaign, and tobacco use by friends and family. Only the oldest participants did not include tobacco users as a tobacco industry marketing influence; however, they had been in the program for a shorter period of time than the other participants. This finding is congruent with adolescent development studies' findings that the complex cognitive processes are somewhat limited until the prefrontal lobe is fully developed in that late stage of adolescence, around 18–25 years of age, suggesting that older adolescents may have an easier time understanding a third-person perspective, which is important to recognizing marketing practices [22]–[23]. As reflected in this study, the participants might understand the underlying concepts of tobacco industry marketing; however, distinguishing tobacco industry marketing influences from individual tobacco use, in alignment with the program, would be more challenging.

Program materials, team processes, available time, community connection, and resources were mentioned by the participants as factors that influenced activity selection and participation. Yet, only a few of the oldest participants took part in the program's main activities that confronted the influence of POS tobacco advertisements or held meetings with community officials to promote policy change. Perhaps this implies that these oldest focus group participants had the ability to be change agents due to their greater cognitive maturity and participation in activities that require pragmatic reasoning or strategic thinking, skills that are different than the ability to plan an event [24]. Additionally, some studies have shown that personal confidence or self-efficacy, which can differ due to age or cognitive development, may influence youth participation [25]; on the other hand, a 2012 evaluation of new tobacco policy campaign materials for Oklahoma's youth program found that increased youth confidence to advocate for local policy did not spur increased advocacy behavior, at least in the short term [26]. As found in another study, adolescents who were younger may have felt empowered to make a difference; thus they joined and stayed active in the program [14]. This study found that participants who wanted to prevent younger kids from starting tobacco use or to help people to quit using tobacco had perceptions of tobacco industry marketing influences that included individual tobacco use. They also were involved with health education and cessation promotional activities. Since youth are more influenced by the “gut reactions” and the sensation of pleasure and reward during this stage of cognitive adolescent development, these findings are not out of place [23].

Others have found that youth-adult partnerships have been difficult to operationalize due to the realities of age-related development and the capacities of the youth within organizational, cultural, and societal contexts [27]. Moreover, some programs have noted time constraints or youth needing assistance to see the social contexts behind the problem [26], [28]. Such studies have hypothesized that additional time was needed for the youth programs to achieve the defined outcomes; though our study found that time was a barrier to the Oklahoma youth program, it also found that the understanding of tobacco industry marketing and participation in activities that addressed POS tobacco advertisements and tobacco sales at retail stores were related to the age of the youth and not the length of time the youth were in the program. A quantitative study of Oklahoma youth empowerment program's staff supports the idea of age-related differences in team activities. The study found that 50% (10/20) of the teams with youth who were less than 13 years old engaged in policy or tobacco industry marketing type activities compared to 92% (34/37) of teams with youth 13 years and older [29].

There is power in youth sharing their experiences and calling for policy changes through their own stories and voices [13]. Such changes can happen when facilitated by programs that help youth develop and express their desires to improve their environment [30]. This study provided insight into the importance of age-appropriate communication to develop a mutual understanding between program staff and youth members. Others have noted that word choice, reframing, and the particular community context influence the effectiveness of youth empowerment programs [30]. Program and policy change movements must exercise discretion to align strategies and outcomes with adolescent development so that youths' intentions, actions, and voices represent their own perspectives [31]. For meaningful and effective engagement with youth, it is recommended that the program constantly seek input from youth to tailor programming that resonates with their evolving perspectives, interests, and abilities, perhaps with an emphasis on the sensation of reward and “gut” reactions for younger youth.

This study was limited by its cross-sectional design and its reliance on self-reported data collected in 2008. Absent from the focus groups were youth who were not regularly active with the team. Such team members may have expressed different perceptions, motivations, and participation, as shown by previous research [21]. Also, those who were less than 13 years of age may have different viewpoints than those who participated. Additional studies are encouraged to explore with youth of varying ages and participation levels on factors that affect their perceptions of the program, activity and participation choices, and industry marketing influences.

## 5. Conclusion

This study found that the understanding of the program's mission varied by the youths' age and not the length of time in the program. The youth members demonstrated various perceptions of the program and of the tobacco industry marketing influences, which influenced their selection of and participation in activities to support the program's mission. Constantly seeking input from youth to understand and tailor the program design and implementation based upon youths' age is essential to maximizing youths' potential and contribution as truly empowered change agents. These findings may not be limited to tobacco control youth empowerment programs. Lessons learned from this study can inform the development of other youth programs that counter tobacco, food, and beverage industry marketing and address chronic disease prevention within the retail environment.
